# Machine learning for modeling animal movement

**DOI:** 10.1371/journal.pone.0235750

**Published:** 2020-07-27

**Authors:** Dhanushi A. Wijeyakulasuriya, Elizabeth W. Eisenhauer, Benjamin A. Shaby, Ephraim M. Hanks

**Affiliations:** 1 Department of Statistics, Pennsylvania State University, University Park, State College, PA, United States of America; 2 Department of Statistics, Colorado State University, Fort Collins, CO, United States of America; Newcastle University, UNITED KINGDOM

## Abstract

Animal movement drives important ecological processes such as migration and the spread of infectious disease. Current approaches to modeling animal tracking data focus on parametric models used to understand environmental effects on movement behavior and to fill in missing tracking data. Machine Learning and Deep learning algorithms are powerful and flexible predictive modeling tools but have rarely been applied to animal movement data. In this study we present a general framework for predicting animal movement that is a combination of two steps: first predicting movement behavioral states and second predicting the animal’s velocity. We specify this framework at the individual level as well as for collective movement. We use Random Forests, Neural and Recurrent Neural Networks to compare performance predicting one step ahead as well as long range simulations. We compare results against a custom constructed Stochastic Differential Equation (SDE) model. We apply this approach to high resolution ant movement data. We found that the individual level Machine Learning and Deep Learning methods outperformed the SDE model for one step ahead prediction. The SDE model did comparatively better at simulating long range movement behaviour. Of the Machine Learning and Deep Learning models the Long Short Term Memory (LSTM) individual level model did best at long range simulations. We also applied the Random Forest and LSTM individual level models to model gull migratory movement to demonstrate the generalizability of this framework. Machine Learning and deep learning models are easier to specify compared to traditional parametric movement models which can have restrictive assumptions. However, machine learning and deep learning models are less interpretable than parametric movement models. The type of model used should be determined by the goal of the study, if the goal is prediction, our study provides evidence that machine learning and deep learning models could be useful tools.

## 1 Introduction

Many of the parametric animal movement models present in literature, including state space models, stochastic differential equations model, step lengths and turning angles models, and step selection function models [1–4], are geared towards inference rather than predicting realistic animal movement. Predicting animal behavior and movement is important for several reasons. Realistic predictions could aid in the formation of conservation strategies to combat the decline in bio-diversity. For example, predicting how movement patterns will be altered when a new road is built can inform decision makers on selecting the least disruptive route. Predicting movement is also important in the context of understanding the spread of infectious diseases through animal populations. Many diseases are spread through direct contact between individuals. Realistic predictions of the movement of infected individuals can suggest interventions that will optimally alleviate further spread of a disease.

Machine Learning (ML) and Deep Learning (DL) methods are commonly used for predictive modelling in many fields like artificial intelligence [5], image processing [6], neuroscience [7], genomics [8] and more. However, they have seldom been used for modeling or predicting animal movement. In this study we explore the use of a range of machine learning/deep learning tools to build predictive models for individual and collective movement of ants.

Several machine learning methods have also previously been used for predicting animal behavior in cheetahs [9] and penguins [10]. [11] compared the use of 5 algorithms—support vector machines, random forests, linear discriminant analysis and artificial neural networks—to predict different behavioral modes in vultures. [12] use deep neural networks to predict non-diving and diving behavior in shags, guillemots and razorbills using GPS data. They found that their method did better than hidden Markov models (HMMs) at prediction. [13] used Random Forests to study tree swallow occurrence in North America. All these studies predict animal behaviour which is framed as a binary or multi-class classification problem. In this work we develop an approach to use machine learning methods to not only predict behaviour but also actual movement trajectories.

Most studies of animal movement use parametric models, with the goal of understanding how and when animals move the way the do. State space models like HMMs have been used widely for understanding animal behavior [14–16]. Step selection function models are a class of animal movement models that focus on the analysis of species-habitat associations [1, 17]. Resource selection models use a weighted distribution formulation of a point process model to model individual independent movements. These models provide insight on the animal’s preference given its environment [2, 18, 19].

Another common approach to modeling movement is a class of models based on “step lengths” and “turning angles” [3, 20]. A movement is defined by the straight line distance between two consecutive locations (step length) and the turning angle between three consecutive locations. Each step and turn is assigned to one of a number of random walks where each is characteristic of a different behavioral state. [21] used these methods to model group dynamic movement. This was based on a framework with a group level model that describes the group’s center and an individual level model that describes the individual animal’s movement relative to the group center. Multi-state random walks were used in both levels of the framework. [22] extended these models to continuous time using a joint bearing and speed process with parameters dependent on a continuous time behavioral switching process.

Stochastic differential equation models are another type of animal movement model that has been used to study the interaction between animals and their habitat. [23] used a stochastic differential equation model to study the influence of roads and grassland foraging areas on elk movements. [24] used a potential surface for inference on regions of attraction and repulsion on the space-time surface that the elk are moving on. [4] used a spatially varying stochastic differential equation model to obtain insight into the spatial structure of ant movement in the ant nest. All of these parametric models make strict assumptions about the underlying processes. While making these assumptions allows for easy interpretation of results, such as associations between movement and habitat, if the modeling assumptions are imprecise, the resulting model may have poor predictive power.

In this study we use machine learning methods to classify animal movement behaviour and also predict movement paths that will enable us to build stochastic movement generators. These are useful in scenarios where collecting actual movement data is laborious. It can be a useful component in simulating the spread of a disease through an animal population and can also be a useful tool in suggesting improvements to a system. Previous studies that used machine learning methods for modeling animal movement only built individual level models. In this study we look at modeling collective animal movement in addition to individual level movement.

Ant colonies are self-organized complex social systems. They are a social insect species that have been used to study infectious disease dynamics [25]. We use ant colony data and our main goal is to build realistic movement simulators. This is with the eventual aim of simulating in silico ant colonies to aid in hypothesis generation and complement experimental work. As predicting ant movement is important when building simulators, we look at one step ahead predictions to gauge the accuracy of the models. We also look at 1000 steps ahead simulations to see if simulations based off of machine learning methods can capture general movement patterns exhibited in the actual data. We compare several machine learning and deep learning methods as well as a stochastic differential equation model that has been previously used in [4] to model this ant colony. In order to demonstrate the generalizability of this framework we also use it to model migratory movement of lesser black-backed gulls *Larus fuscus*. Here we do one step ahead predictions and simulate short migratory paths for illustrative purposes.

## 2 Materials and methods

### 2.1 Ant movement data

**Fig 1:**
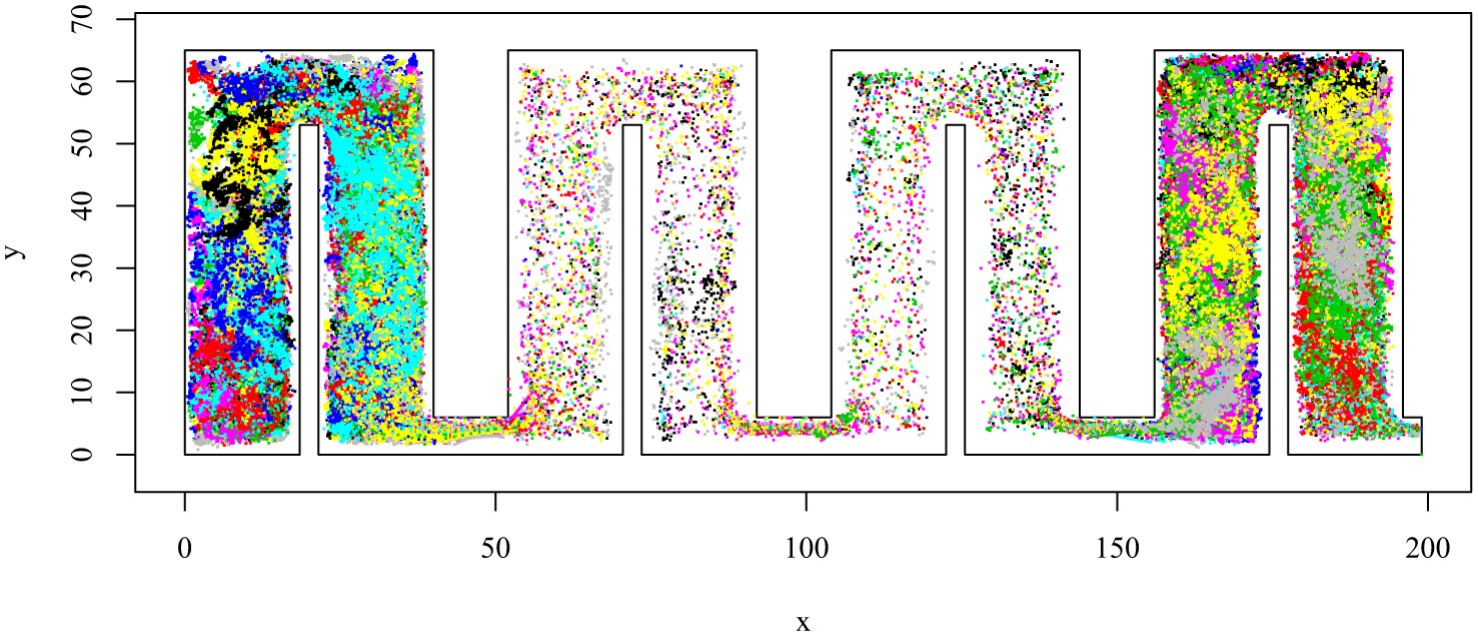
Visualization of the ant movement data colored by each unique ant identifier. Ants mostly reside in Chambers 1 and 4 and seem to have faster more directed movements through Chambers 2 and 3.

We use ant data from the Hughes Lab at Penn State. This data consists of the movement of 73 common black carpenter ants (Camponotus pennsylvanicus) in a custom constructed nest. The nest has four chambers, with each chamber divided into two parts by a barrier in the middle. There is a narrow passageway between the two halves of each chamber. The total nest size is 65mm by 160mm and each chamber measures 65mm by 40mm. There are doorways in between chambers that measure 6mm across, and an exit from the nest in the 4th chamber (the far right in Fig 1). For a full description of the data collection, and original goals for this data, see [26].

The raw data consist of 2-dimensional location coordinates for each ant j at each time point i. Observations were made every second and there are 14400 observations amounting to 4 hours of movement data. Each ant has a unique identifier and a human observer recorded their position by clicking on their location using a custom software package. The velocity of movement in the x and y directions are then approximated using first differences. This dataset is unique in having location data at a very fine temporal resolution for each individual of the colony with no missing data and very little measurement error. A more detailed description of the data collection process can be found in [26] and [4].

A plot of all the data is given in Fig 1, with recorded locations of each individual ant given by a unique colour. Visually it is evident that the ants predominantly occupy chambers 1 and 4 while moving fast through Chambers 2 and 3.

The data can be accessed at https://doi.org/10.5061/dryad.sh4m4s6 as supplementary material to [26]. The low density tracking data of colony 1 is used in this study.

#### 2.1.1 Derived variables

We consider supervised learning approaches to animal movement, and seek to predict future movement with characteristics of the previously observed movement behavior. We used the raw data to derive new variables that capture previous behaviour of individual ants as well as movement of neighbouring ants with the aim of informing predictions. We created 39 variables for each ant at each time step. These variables are meant to capture a wide range of potential movement behavior. A complete list of variables is given in Table 1. The motivation behind deriving these variables are threefold. First to capture previous behaviour of the individual ant. This is captured by the variables *x*_*t*−1:*t*−5_,*y*_*t*−1:*t*−5_, *vx*_*t*−1:*t*−5_,*vy*_*t*−1:*t*−5_, Stationary time and *d*_*t*−1_. The second to capture the interaction between the ant and the structure of the nest. The variables that capture this are Sub chamber number and distance to walls in North, South, East and West directions. The rest of the variables capture the behavior of neighboring ants. The derived variables were selected by visually observing the movement of the ant colony through time as well as referencing [4]. To be concise, we will denote the vector of all derived variables at time *t* as **u**_*t*_.

$$\begin{array}{c} {\text{u}}_{t-1}=\left(x_{t-1:t-5},y_{t-1:t-5},vx_{t-1:t-5},vy_{t-1:t-5},\text{Other}\;\text{derived}\;{\text{variables}}_{t-1}\right) \end{array}$$ (1)

**Table 1:** List of derived variables.

Variable	Description
*x*_*t*−1:*t*−5_,*y*_*t*−1:*t*−5_	Lagged (*x*, *y*) co-ordinates upto 5 lags
*vx*_*t*−1:*t*−5_,*vy*_*t*−1:*t*−5_	Lagged *x*, *y* velocities for upto 5 lags
Stationary time	Number of consecutive time points the ants were stationary. See Fig 2b for stationary time for ant ID 397
*d*_*t*−1_	Distance traveled in the penultimate time step
Sub chamber	Sub chamber number. Each chamber is separated into two halves with the wall in the middle. Sub chambers are numbered from 1-8 from left to right.
Dist to wall in N, S, E, W directions	Distances from the ant’s current position to the nearest wall in the North, South, East and West directions
Distance to nearest neighbor (nn)	Distance from the ant’s current position to the nearest ant
*nn*_*xt*−1_,*nn*_*yt*−1_	(*x*, *y*) location of the nearest neighboring ant at time *t*−1
*nn*_*vxt*−1_,*nn*_*vyt*−1_	Velocities in the (*x*, *y*) directions of the nearest neighbouring ant at time *t* − 1
Q1, Q2, Q3, Q4	Number of ants in each quadrant. Quadrants 1, 2, 3 and 4 are defined as 8x8 squares around the ant as given in Fig 2a. This variable captures the density of neighboring ants in different directions around each ant.
Number of neighboring ants who are still	Number of ants in a 10mm radius around the ant that are still at *t* − 1
Number of neighboring ants who are moving	Number of ants in a 12mm radius around the ant that are moving at *t* − 1
Distance to the queen	Distance from the ant to the queen. The queen has the ID “Que”.

**Fig 2:**
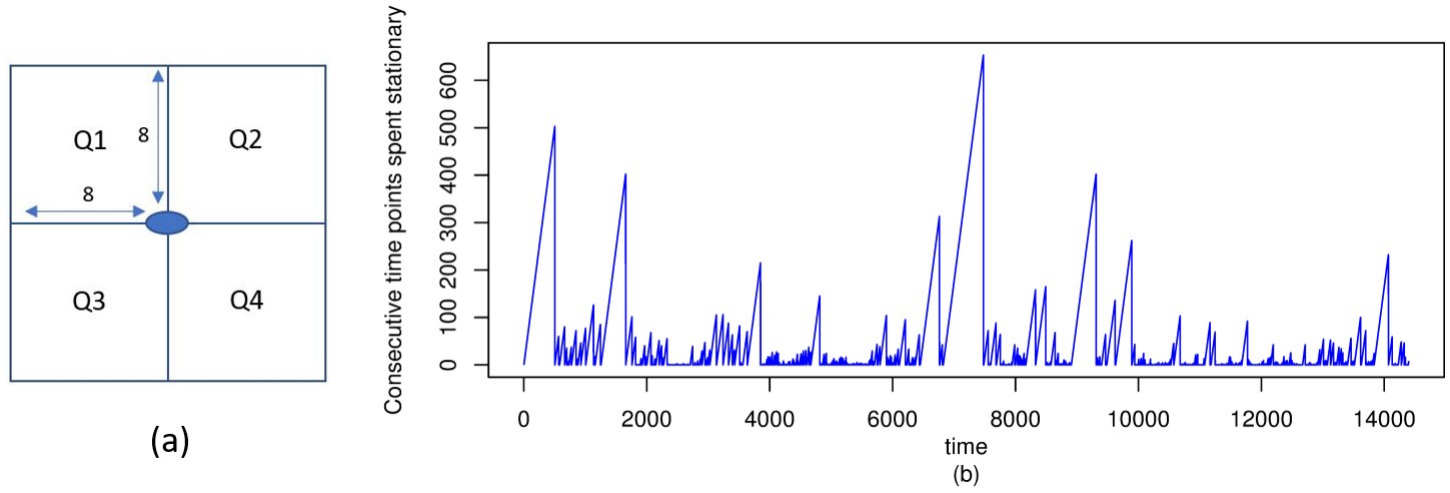
Stationary time and definition of 4 quadrants. (a) gives a graphical description of the 4 quadrants around each ant. Each quadrant has side length 8mm. (b) gives the plot of stationary time for ant ID 397 and shows that there are long periods of no movement.

### 2.2 Migratory gulls data

**Fig 3:**
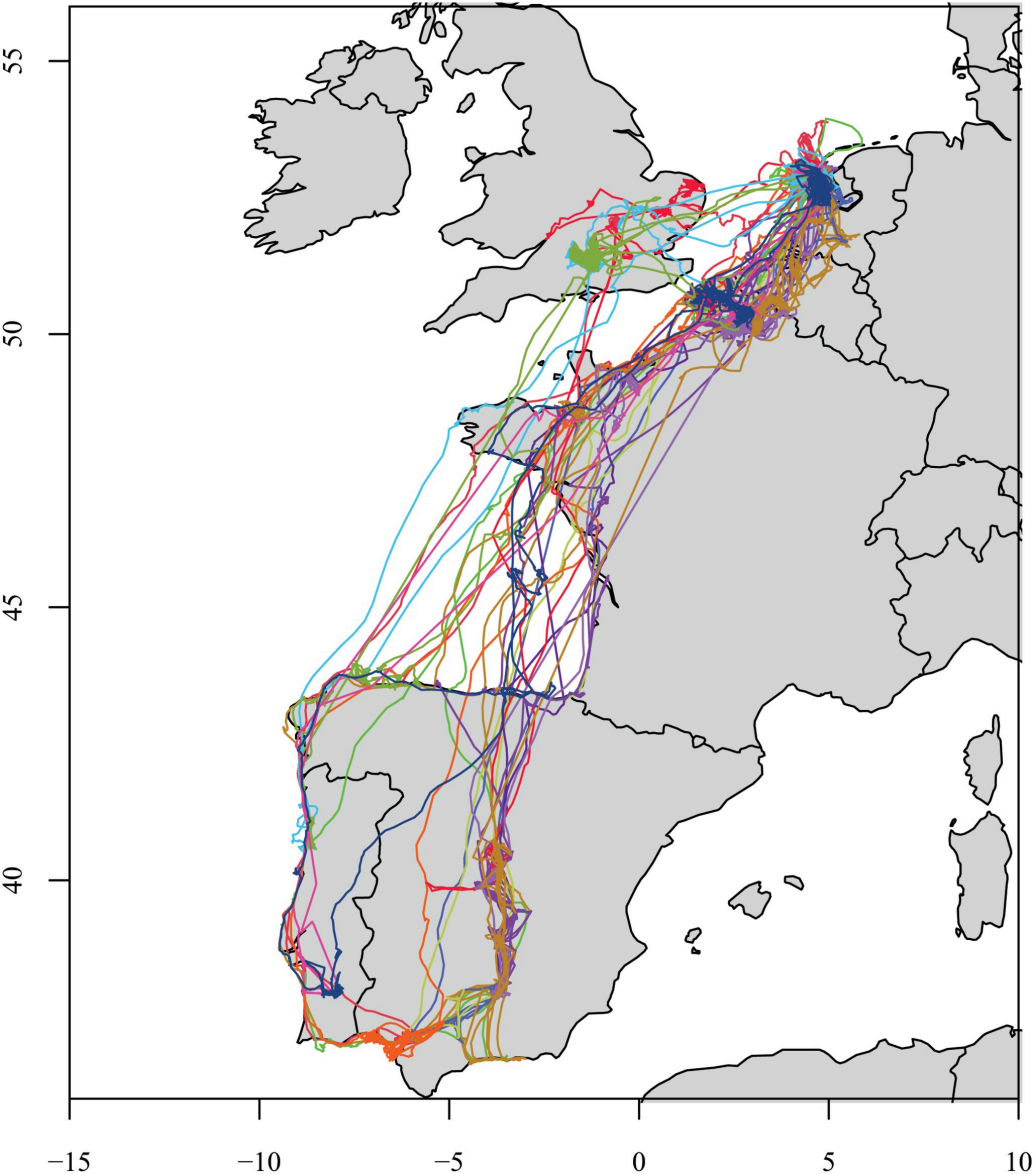
Migratory paths of 15 gulls years. Each differently colored line denotes an individual gull year. All gulls migrate southbound to the Iberian Peninsula.

For a second example system, we use 15 bird years of migratory paths of lesser black-backed gulls (*Larus fuscus*). This data [27] was obtained from supplemental information from [28]. These 15 paths come from 7 individual gulls over the time period 2010-2014. All paths have the same migratory strategy of wintering in the Iberian Peninsula. Fig 3 gives the paths of all 15 bird years. For each bird year, tracking data was obtained every 20-30 minutes. See [28] for more details about the data collection process. Some bird years had missing values and we used linear interpolation for imputation of missing data.

Looking at the Fig 3 we can see four distinct behavioral states, which we define based off of location and movement patterns: Northern range, Southbound migration, Southern range and Northbound migration. We classified each observation as being in one of these states as follows. All birds began in the Northern range state. A bird switches to the Southbound migration state when their movement track covers over 0.91 degrees Latitude in the course of a ten-hour period, and stays in that state until they do not cover at least 0.91 degrees Latitude in a Southbound direction, in which case they are then classified as being in their Southern range. Birds stay in their Southern range until their movement track covers over 1.0 degrees Latitude in a northbound direction in the course of a ten-hour period, and stay in this state until the do not cover at least 1.0 degrees Latitude in a Northbound direction. Occasionally, birds made stopovers along their migratory route. In this case, we classified birds as being in either the Northern range or Southern range state depending on whether the stopover site was closest to the farthest North or farthest South observed location of the bird that year. The result was a behavioral state for each bird at each time point in our study.

In this dataset, location was given as latitude and longitude coordinates. We first converted these into UTM coordinates before carrying out our analysis. Similar to the ant dataset, we computed derived variables to capture features of the gulls movement. The derived variables are lagged locations in *x* and *y* directions, lagged velocities in *x* and *y* directions, day number (number of days since 1st of June each year), time of day (decimal number between 0 and 1) and distance travelled in the penultimate time step.

Unlike the ant movement data we do not have colony level data for the gulls and therefore only aim to model individual movement of gulls in this study.

### 2.3 Modeling individual and collective movement

Let **p**_*i*,*t*_ be the *x*, *y* locations of animal *i* at time *t*. The models used in this study can be divided into two main types.

1. Independent Models: Each animal is independently and identically distributed: **p**_*i*,*t*_ = (*x*_*i*,*t*_, *y*_*i*,*t*_) ∼ *g*(**u**_*i*,*t*−1_)
2. Joint Models: All animals are modeled jointly: $P_{t}=\left(\begin{array}{c} p_{1,t}{}^{T}\\ p_{2,t}{}^{T}\\ \vdots \\ p_{N,t}{}^{T} \end{array}\right)∼g\left(u_{1,t-1},\cdots ,u_{N,t-1}\right)=g\left(u_{t-1}\right)$

**P**_*t*_ is the joint distribution of locations of all animals in a colony or herd at time *t*. *g* is a probability model yet to be specified.

We use both types of models to generate colony simulations after model fitting for the ant movement data. In order to demonstrate the generalizability of our framework to other species we use the individual level model to simulate migratory paths for the gulls data. We do not use the colony level model since colony level data were not available, with only a small sample of birds (seven individuals) observed. When using individual movement models we model each animal using the same model. This approach assumes that one model is sufficient for modeling an individual animal and that the underlying process that determines behavior is unchanged from one animal to another. Thus, individual animal models capture population-level average behavior, while the colony level models have more flexibility in capturing behavioural patterns specific to each individual animal as well as interactions between the animals.

### 2.4 A general framework for modeling high-resolution animal movement

We now present a general framework for modeling animal movement. This framework allows for state-switching, with different movement behavior in each state, and is flexible enough to allow for parametric or nonparametric approaches for movement in both the state-switching and movement behavior.

On average, ants in our data are stationary 76% of the time. These ants are moving inside the nest for 22% of the time, and are outside of the nest (in a feeding arena) for 2% of the data. Positions of ants outside the nest are not recorded; video tracking of these ants was only done inside the nest. Following [4], we view these three distinct states the ant could be in (moving within the nest, stationary within the nest, and outside of the nest) as behavioral states. It has long been recognized that, when movement is observed at high temporal resolution, that animals of most species exhibit similar state switching behavior [3]. The most common approach used to model state-switching in animal movement is through hidden Markov models. Here, we consider the case where the temporal resolution is fine enough, and the measurement error on the animal locations is small enough, that it is reasonable to assume that an animal’s behavioral state is evident at each time point from the position data themselves. When this is not the case, the following framework could be used as a model for latent animal movement and state switching.

We assume in general that animal *i* is in behavioral state *m*_*it*_ at time *t*, with *m*_*it*_ ∈ {1, 2, …, *M*}. For the ants, we follow [4] and assume that *M* = 3, with

$$\begin{array}{c} m_{it}=\{\begin{array}{cc} 0 & \;\text{if}\;\text{ant}\;\text{does}\;\text{not}\;\text{move}\\ 1 & \;\text{ant}\;\text{moves}\;\text{out}\;\text{of}\;\text{the}\;\text{nest}\\ 2 & \text{ant}\;\text{moves}\;\text{within}\;\text{the}\;\text{nest} \end{array} \end{array}$$

For the gulls we assume that *M* = 4, with

$$\begin{array}{c} m_{it}=\{\begin{array}{cc} 1 & \text{gull}\;\text{stays}\;\text{in}\;\text{the}\;\text{Northern}\;\text{range}\;\text{(Scandinavia}\;\text{and}\;\text{Great}\;\text{Britain)}\\ 2 & \text{gull}\;\text{migrates}\;\text{southbound}\;\text{to}\;\text{the}\;\text{Iberian}\;\text{Peninsula}\\ 3 & \text{gulls}\;\text{stays}\;\text{in}\;\text{the}\;\text{Southern}\;\text{range}\;\text{(Iberian}\;\text{Peninsula)}\\ 4 & \text{gull}\;\text{migrates}\;\text{northbound}\;\text{to}\;\text{the}\;\text{Northern}\;\text{range} \end{array} \end{array}$$

In general, we assume that an animal’s movement behavior is different for each behavioral state. We thus propose a general framework for the analysis of animal movement data in which an observed behavioral state process {*m*_*it*_, *t* = 1, 2, …, *T*} is modeled and predicted using a classification model, and bivariate movement {**p**_*it*_, *t* = 1, 2, …, *T*} is modeled using a continuous-valued response model. Thus, we propose that at time *t* in this general framework can be expressed as follows. The state process at time *t* is modeled using a classification model:

$$\begin{array}{c} P\left(m_{it}=k|\left\{m_{is},{\text{p}}_{is},s\in 1,2,\ldots ,t-1\right\}\right)=f_{m_{k}}\left({\text{u}}_{t-1}\right) \end{array}$$ (2)

and, conditioned on this behavioral state, we model the animal’s discrete-time velocity **v**_*it*_ = (**p**_*it*_ − **p**_*it*−1_)/*δt* as

$$\begin{array}{c} {\text{v}}_{it}|\left(m_{it}=k\right)=f_{vk}\left({\text{u}}_{t-1}\right)+\epsilon_{it},\;\epsilon_{it}∼h_{m_{it}}. \end{array}$$ (3)

Here **f**_*vk*_ is a vector-valued function which controls the mean movement of the animal in state *k*. Similar formulations have been proposed by [29], who let **f**_*vk*_ be the negative gradient of a potential surface, and [4, 30], who consider functions resulting from numerical approximations to stochastic differential equation models for animal movement. The framework we propose is flexible enough to encompass most existing parametric models for animal movement, as well as nonparametric approaches. For example, **f**_*vk*_ could be the prediction from a bivariate random forest [31] or neural network predictor trained on movement data, and $f_{m_{k}}$ could be the predicted probability of transitioning to state *k* from a neural network classification algorithm.

When applying this framework to the ant movement data, we have *m*_*it*_ = 0 for a stationary ant. Since the ant does not move the discrete time velocity should be 0, i.e. *f*_*v*0_ = 0 and *ϵ*_*it*_ = **0**. The location of ants when they move out of the nest is not recorded. Therefore we assign their location to be (199, 0) which is the bottom right most coordinate of the nest at the opening of the nest to the feeding area. Therefore, for an ant that moves out of the nest *m*_*it*_ = 1, we adjust (*x*_*t*_, *y*_*t*_) to be (199, 0). Since we now have (*x*_*t*_, *y*_*t*_) and (*x*_*t*−1_, *y*_*t*−1_) we can calculate the discrete time velocity to be *f*_*v*1_ = (199 − *x*_*t*−1_, 0 − *y*_*t*−1_) and *ϵ*_*it*_ = **0**. When an ant moves within the nest, i.e. *m*_*it*_ = 2 we fit a bivariate continuous response model using all the movements that resulted in an ant moving. This is difficult to do for colony level models as we cannot dynamically alter the output dimensions based on which ants are moving and which are not. In this case, we use all movement data to model velocity and use the output of (1) to zero out velocities of ants that are classified as not moving or are outside of the nest. When *m*_*t*_ = 1 (ant has moved out of the nest) we adjust (*x*_*t*_, *y*_*t*_) to be (199, 0). This is the bottom extreme right corner of the nest where the feeding area is located. We do not predict **v**_*t*_ but rather calculate **v**_*t*_ = (199 − *x*_*t*−1_, 0 − *y*_*t*−1_).

When using this framework to model migratory movement of gulls we express **v**_*it*_ and **p**_*it*_ as follows.

$$\begin{array}{c} {\text{v}}_{it}|\left(m_{it}=k\right)=f_{vk}\left({\text{u}}_{t-1}\right)+\epsilon_{it},\;\epsilon_{it}∼h_{m_{it}} \end{array}$$

$$\begin{array}{c} {\text{p}}_{it}\left|\left(m_{it}=k\right)={\text{v}}_{it}\right|\left(m_{it}=k\right)+{\text{p}}_{it-1},\;k=1,2,3,4 \end{array}$$

We have one classification model of the form in Eq (2) and four velocity models, one for each individual state.

In general, the individual level model can be formally expressed as in Eqs (2) and (3). We formally define the colony level model for *N* ants as follows. We denote vector valued variables in bold. We use lower case to denote individual ants and upper case to denote collective ants. **P**_*t*_, **V**_*t*_ and **M**_*t*_ denote colony level locations, velocities and movement behaviours respectively.

$$\begin{array}{c} {\text{P}}_{t}={\left[{\text{p}}_{1t}^{\prime }\;\;{\text{p}}_{2t}^{\prime }\;\;\ldots {\text{p}}_{Nt}^{\prime }\right]}^{\prime },\\ V_{t}={\left[{\text{v}}_{1t}^{\prime }\;\;{\text{v}}_{2t}^{\prime }\;\;\ldots {\text{v}}_{Nt}^{\prime }\right]}^{\prime },\\ M_{t}={\left[m_{1t}\;\;m_{2t}\;\;\ldots m_{Nt}\right]}^{\prime }, \end{array}$$

The colony level model for movement behaviour is of the following form

$$\begin{array}{c} M_{t}|U_{t-1}∼\text{Categorical}\left(F_{m}\left(U_{t-1}\right)\right) \end{array}$$ (4)

The colony level model for velocity is of the following form

$$\begin{array}{c} {\text{V}}_{t}|{\text{U}}_{t-1}={\text{F}}_{v}\left({\text{U}}_{t-1}\right)+\Phi_{t}^{v} \end{array}$$ (5)

$$\begin{array}{c} \Phi_{t}^{v}=\left[\epsilon_{1t\prime }^{v}\;\;\epsilon_{2t\prime }^{v}\;\;\ldots \epsilon_{Nt\prime }^{v}\right]\prime \end{array}$$

We use **F**_*vt*_ for ease of notation to demonstrate how the colony level movement behaviour and velocity models are combined.

$$\begin{array}{c} {\text{F}}_{vt}\left({\text{M}}_{t},{\text{U}}_{t-1}\right)={\left[f_{vm_{1,t}}^{\prime }\left({\text{U}}_{t-1}\right)\;\;f_{vm_{2t}}^{\prime }\left({\text{U}}_{t-1}\right)\;\;\cdots f_{vm_{Nt}}^{\prime }\left({\text{U}}_{t-1}\right)\right]}^{\prime }, \end{array}$$

The colony level model for location is given as follows

$$\begin{array}{c} {\text{P}}_{t}|{\text{U}}_{t-1},{\text{M}}_{t}={\text{P}}_{t-1}+{\text{F}}_{vt}\left({\text{M}}_{t},{\text{U}}_{t-1}\right)+\Phi_{t}. \end{array}$$ (6)

$$\begin{array}{c} \Phi_{t}=\left[\epsilon_{1t}^{(m_{1t})\prime }\;\;\epsilon_{2t}^{(m_{2t})\prime }\;\;\ldots \epsilon_{Nt}^{(m_{Nt})\prime }\right]\prime . \end{array}$$

We apply the above framework to model the entire colony of ants jointly. The colony has 73 individual ants, therefore *N*_*t*_ = 73. To better understand the form of **F**_*vt*_ when this framework is applied to ant colony data we illustrate the form of ${\text{f}}_{vm_{it}}\left({\text{U}}_{t-1}\right)$.

$$\begin{array}{c} {\text{f}}_{vm_{it}}=\{\begin{array}{cc} \left(0,0\right) & m_{it}=0\\ \left(199-x_{i,t-1},0-y_{i,t-1}\right) & m_{it}=1\\ {\text{v}}_{it} & m_{it}=2 \end{array} \end{array}$$

The reasoning behind the form when *m*_*it*_ = 0 and *m*_*it*_ = 1 for the ant colony data is the same as under the individual level model.

The relationship between the error terms $\Phi_{t}^{v}$ and Φ_*t*_ when the colony level model is applied to ant movement data can be illustrated through the relationship between $\epsilon_{it}^{v}$ and $\epsilon_{it}^{m_{it}}$.

$$\begin{array}{c} \epsilon_{it}^{m_{it}}=\{\begin{array}{cc} \left(0,0\right) & m_{it}=0\\ \left(0,0\right) & m_{it}=1\\ \epsilon_{it}^{v} & m_{it}=2 \end{array} \end{array}$$

In specifying the two step model above we have used *f*_*m*_, *f*_*v*_, **F**_*m*_ and **F**_*v*_ as general functions. The form of these functions will vary depending on the machine learning or deep learning methods used.

### 2.5 Machine learning and deep learning methods

There were several factors that influenced our decision on the types of machine learning and deep learning methods to use in estimating *f*_*m*_ and *f*_*v*_. For *f*_*m*_ we consider classification methods that allow for multiple classes (three classes in our case) and can be extended to the multivariate case. A univariate classifier is sufficient for modeling individual ant behaviour while a multivariate extension is needed to model colony level behaviour with the dimensionality of the classifier equal to the number of ants in the colony.

Animal movement is inherently stochastic in nature and so we are interested in obtaining stochastic simulations of movement. Therefore, for estimating *f*_*v*_ we consider methods that allow for sampling a velocity from a distribution of velocities. For ease of implementation we restrict ourselves to classes of models that are capable of estimating discrete categorical responses and continuous multivariate responses.

We consider random forests and neural networks as candidate methods for modeling *f*_*m*_ and *f*_*v*_ at the individual and colony level. We use univariate and multivariate random forests, as well as neural networks, to model *f*_*m*_ and *f*_*v*_ in the individual and colony level models.

#### 2.5.1 Random forests

We here provide a description of the Random Forest algorithm, which we use as one possible machine learning approach for modeling animal movement. Classification and regression trees (CART), the building blocks of Random Forests, were developed by [32]. The CART framework consists of 4 components.

1. A set of binary questions based on the predictors that partition the predictor space.
2. An impurity measure. In this study for classification we use the gini index and for regression we use a measure based on the response variance.
3. A split function: A split is made at each node that results in children nodes. A split function is used to determine the best split. This incorporates the impurity measure and determines the optimal split that results in the children nodes being the most homogeneous among competing splits. Nodes that do not have children nodes are called terminal nodes or leafs.
4. A way of determining the tree size.

In a univariate response setting with predictors *x*_*ij*_ and responses *y*_*i*_ where (*i* = 1, …, *n*;*j* = 1, …, *p*), with *n* and *p* being the total number of samples and the number of predictor respectively, let us consider the data at node *m* to be *Q*. Let *θ* = (*j*, *t*_*m*_) be a candidate split consisting of a predictor j and a threshold *t*_*m*_ that partitions the data into subsets *Q*_*left*_(*θ*) and *Q*_*right*_(*θ*) defined as

$$\begin{array}{c} Q_{left}\left(\theta \right)=\left(x_{ij},y_{i}\right)\in Q|x_{ij}<=t_{m} \end{array}$$

$$\begin{array}{c} Q_{right}\left(\theta \right)=Q\backslash Q_{left}\left(\theta \right) \end{array}$$

The impurity at node *m* is calculated using the impurity function *H*(). For a classification problem we use the Gini index defined as

$$\begin{array}{c} H\left(Q\right)=\sum_{k=1}^{K}p_{mk}\left(1-p_{mk}\right) \end{array}$$

$$\begin{array}{c} p_{mk}=\frac{1}{N_{m}}\sum_{i\in Q}I\left(y_{i}=k\right) \end{array}$$

Where *k* = 1, …, *K* and K is the number of classes for the categorical response *y*_*i*_. *i* ∈ *Q* represents the subset of all samples in node *m*. |*i* ∈ *Q*| = *N*_*m*_.

In the regression (movement) setting we use mean squared error defined as

$$\begin{array}{c} H\left(Q\right)=\frac{1}{N_{m}}\sum_{i\in Q}{\left(y_{i}-{\bar{y}}_{m}\right)}^{2} \end{array}$$

$$\begin{array}{c} {\bar{y}}_{m}=\frac{1}{N_{m}}\sum_{i\in Q}y_{i} \end{array}$$

*i* ∈ *Q* and *N*_*m*_ have identical meaning as before. We calculate the splitting rule at node *m* by selecting the split that minimizes the impurity.

$$\begin{array}{c} G\left(Q,\theta \right)=\frac{n_{left}}{N_{m}}H\left(Q_{left}\left(\theta \right)\right)+\frac{n_{right}}{N_{m}}H\left(Q_{right}\left(\theta \right)\right) \end{array}$$

$$\begin{array}{c} \theta^{*}={\text{argmin}}_{\theta }G\left(Q,\theta \right) \end{array}$$

*n*_*left*_ = |*Q*_*left*_(*θ*)|, *n*_*right*_ = |*Q*_*right*_(*θ*)|. This is repeated until the maximum allowable depth of the tree is reached [33].

Extending CART to the multivariate case with *n*_*o*_ responses *y*_*ij*_ (*i* = 1, …, *n* and *j* = 1, …, *n*_*o*_) can be done simply by adjusting the impurity function H() appropriately. [33] states that this can be done by computing the average impurity across all the outputs. For the classification case this can be formally represented as

$$\begin{array}{c} H\left(Q\right)=\frac{1}{n_{o}}\sum_{j=1}^{n_{o}}\sum_{k=1}^{K}p_{mkj}\left(1-p_{mkj}\right) \end{array}$$

$$\begin{array}{c} p_{mkj}=\frac{1}{N_{m}}\sum_{i\in Q}I\left(y_{ij}=k\right) \end{array}$$

Here we assume that each categorical response variable has the same categories. In our study we use multiple categorical variables to correspond to behaviours of each individual ant in the colony with each these variables taking values of 0, 1 and 2 as defined in (2).

The extension to the regression setting is discussed in detail in [34]. Here a covariance weighted analog is proposed. This is equivalent to adjusting the *H*() as follows

$$\begin{array}{c} H\left(Q\right)=\frac{1}{N_{m}}\sum_{i\in Q}({\text{y}}_{i}-{\bar{\text{y}}}_{m}{)}^{{}^{\prime }}{\text{V}}^{-1}\left(m,\eta \right)({\text{y}}_{i}-{\bar{\text{y}}}_{m}) \end{array}$$

*η* represents parameters that can be used to prescribe covariance structures for e.g. (auto regressive behaviour, compound symmetry) but in this study we consider **V**^−1^(*m*, *η*) to be the identity matrix. In our study we consider the velocities of all ants in an ant colony to be the multivariate continuous responses and the data did not indicate a structure in the covariance matrix that warranted for a specific form for **V**^−1^(*m*, *η*) to be used.

In the univariate case for classification, prediction results in a *K* dimensional vector $\left(f_{m_{0}},f_{m_{1}},\ldots ,f_{m_{K}}\right)={\text{f}}_{m}$ where each element is a proportion of the cases in that leaf that fall into class *k*. The predicted class then is the class associated with $max(f_{m_{0}},f_{m_{1}},\ldots ,f_{m_{K}})$. The multivariate analog is a natural extension where now we have a *K* × *n*_*o*_ matrix and the class corresponding to the maximum for each column is obtained. The resulting will be an *n*_*o*_ dimensional vector with predicted classes for each response.

For the regression case prediction for each leaf is the response means of the cases reaching that leaf. For the multivariate case it is simply the vector response means of the cases.

We use univariate and multivariate random forests to model *f*_*m*_ and *f*_*v*_ in the individual and colony level models. Random Forests [31] are a ensemble of CART trees. Each tree is fit using a bootstrap samples of the data. At each node,*l* predictors are randomly selected (*l* < < *p*) and used for node splitting. Predictions using random forests are made by majority vote of the ensemble learners for classification problems and mean of the ensemble learners for regression problems.

In order to get stochastic simulations, we randomly sample a single learner from the ensemble learners and use its results for both the individual and colony level models.

We use a grid search to tune these models. We used a range of values for the maximum depth of the trees, the minimum number of samples in a leaf and minimum samples needed for a split for tuning. We evaluated each model based on in sample and out of sample performance. The goal was to select a set of hyper-parameters that lead to good performance on both in sample and out of sample data in order to safeguard against overfitting. We implement Random Forests using the sklearn package [33] in Python.

#### 2.5.2 Deep learning methods

In the previous sub-section we described Random forests, which can be used to both predict categorical behavioural states as well as predict movement paths for animals. We now describe deep learning approaches, another popular machine learning approach which can accomplish these same tasks.

Deep learning uses abstract layers of latent variables in a hierarchical structure to perform pattern matching and prediction [35]. It finds a predictor for an output *Y* given a high-dimensional input *X*. This can be represented as an input-output mapping *Y* = *F*(*X*). The multivariate function *F* is a superposition of univariate semi-affine functions. We use two types of neural networks- feed-forward neural network and recurrent neural networks. The difference between them as their names suggests is that recurrent neural networks have recurrent connections that feed previous states as inputs when computing subsequent states. We consider two types of recurrent neural networks- simple recurrent networks and Long Short Term Memory networks [36]. The latter improves on the vanishing gradient problem that is encountered in simple recurrent neural networks and has the ability to retain information for long time intervals. We use truncated Principal Component Analysis as a dimension reduction method for the colony level neural network models.

To predict velocities in the *x* and *y* directions we exploit the ensemble learning property of random forests to sample velocities while we use monte carlo dropout [37] for neural networks that enable us to easily sample from the posterior predictive distribution.

We embed information about previous time points in all our predictive models by including lagged velocities and locations in **u**_**t**_. There are however, variants of neural networks that explicitly model sequence data, like time series of animal movements. Recurrent neural networks (RNN) and Long Short Term Memory (LSTM) [36] models are common deep learning methods used in applications like speech recognition [38], forecasting exchange rates [39], and text classification [40]. We explore these methods for modeling animal movement.

In summary, the methods we pick enable us to

1. Model discrete categorical responses for *f*_*m*_
2. Model continuous multivariate responses for *f*_*v*_
3. Give stochastic simulations

In addition to the above simple recurrent neural networks and LSTM models account for the time series nature of the data.

#### 2.5.3 Feed forward neural networks

Let $f_{l}^{1},f_{l}^{2},\ldots ,f_{l}^{N_{l}}$ be the activation functions associated with the *l*^*th*^ hidden layer. The semi-affine activation rule at the *i*^*th*^ neuron of the *l*^*th*^ hidden layer is given by

$$\begin{array}{c} f_{l}^{i}=f_{l}(\sum_{j=1}^{N_{l-1}}w_{j}z_{j}+b_{li}) \end{array}$$

Where *w*_*j*_ are the weights corresponding to the *N*_*l*−1_ inputs {*z*_*j*_} from the (*l* − 1)^*th*^ layer to the *i*^*th*^ neuron of the *l*^*th*^ layer. *b*_*li*_ denotes the offset or bias sometimes called thresholds or activation levels [35].

Popular choices for the activation function *f*_*l*_ are the sigmoid function, tanh ($tanh\left(x\right)=\frac{sinh(x)}{cosh(x)}=\frac{e^{x}-e^{-x}}{e^{x}+e^{-x}}$), softmax and rectified linear unit (*ReLU*(*x*) = *max*(0, *x*)). This choice is largely governed by the type of layer (hidden or output) and if it is the output, the type of task involved (classification or regression). The goal is to select optimal weights and bias based on a criterion which is often to minimize a loss function. The loss function depends on whether the type of task is classification or regression.

In this study we use MLP as a possible method of approximating *f*_*m*_. For the individual level model we can represent a two-hidden layer MLP with *h*_1_ hidden units in layer 1 and *h*_2_ hidden units in layer 2 in the following form.

$$\begin{array}{c} {\text{z}}_{1,t}=f_{1}\left(W_{1}{\text{u}}_{\text{t}-1}+{\text{b}}_{1}\right) \end{array}$$

$$\begin{array}{c} {\text{z}}_{2,t}=f_{2}\left(W_{2}{\text{z}}_{1,t}+{\text{b}}_{2}\right) \end{array}$$

$$\begin{array}{c} {\text{z}}_{3,t}=W_{3}{\text{z}}_{2,t}+{\text{b}}_{3} \end{array}$$

$$\begin{array}{c} {}[\begin{array}{c} f_{m_{0},t}\\ f_{m_{1},t}\\ f_{m_{2},t} \end{array}]=f_{3}\left({\text{z}}_{3,t}\right)=\text{softmax}\left({\text{z}}_{3,t}\right) \end{array}$$

Where $W_{1}\in R^{h_{1}\times p}$, $W_{2}\in R^{h_{2}\times h_{1}}$,$W_{3}\in R^{3\times h_{2}}$, ${\text{b}}_{1}\in R^{h_{1}}$, ${\text{b}}_{2}\in R^{h_{2}}$ and ${\text{b}}_{3}\in R^{3}$.

In this model *f*_3_ is the softmax function [41] and is applied element-wise on **z**_3_. For the *k*^*th*^ class the softmax function is computed as

$$\begin{array}{c} f_{3}\left({\left({\text{z}}_{3,\text{t}}\right)}_{k}\right)=\frac{\text{exp}({\left({\text{z}}_{3,\text{t}}\right)}_{k})}{\sum_{j=0}^{2}\text{exp}\left({\left({\text{z}}_{3,\text{t}}\right)}_{j}\right)}\;k=0,1,2 \end{array}$$

*f*_1_ and *f*_2_ can activation functions of our choosing. See [42] for a list of activation functions to choose from. In this study we restricted hidden layer activation functions to relu, tanh and elu.

The parameters in the above model *W*_1_, *W*_2_, *W*_3_, **b**_1_, **b**_2_ and **b**_3_ are estimated by minimizing the categorical cross entropy, a popular loss function for multi-class classification problems. [43] specify categorical cross entropy loss as follows.

Let *f*_3_((**z**_**3**,**t**_)_*k*_) = *p*_*k*_, **p** = [*p*_0_, *p*_1_, *p*_2_] and $\sum_{k=0}^{2}p_{k}=1$. If the class of the categorical response variable corresponding to *u* is *k*, we define the one hot encoding to be **e**_*j*_ where *e*_*ji*_ = 1 if *i* = *j*, otherwise 0. One hot encoding replaces a single categorical response variable with 3 binary dummy variables. The loss function is then defined as

$$\begin{array}{c} \text{Loss}=\sum_{k=0}^{2}e_{jk}\text{log}(\frac{1}{p_{k}})=\text{log}(\frac{1}{p_{j}}) \end{array}$$

The loss is minimized using a gradient descent algorithm of our choice. See [42] for a list of optimizers and references for each optimizer.

Overfitting is a concern when deciding the number of hidden neurons foe each layer. Dropout [44] is a technique that is used to alleviate this. This is achieved by removing hidden neurons with a probability p* and then see how this affects the loss function and optimization problem. See [44] for more details.

In addition to the parameters we need to estimate, we also have hyper-parameters which we need to specify by tuning the model. We do a grid search of the hyper-parameter space and use out of sample performance to inform us on choosing the most appropriate values of the hyper-parameters. The hyper-parameters we considered were the number of neurons in each hidden layer, the dropout probability, the activation function of each hidden layer, the number of hidden layers, the optimizer in the tuning process and the learning rate associated with the optimizer. We used accuracy rate as the performance metric.

We use the keras package [42] in Python to fit these neural network and use a grid search in the tuning process.

Extension of this to the colony level model is quite straightforward. The output layer *f*_3_(**z**_3_) was modified as follows

$$\begin{array}{c} \begin{array}{c} {\text{z}}_{3,1,\text{t}}=W_{3,1}{\text{z}}_{2,t}+{\text{b}}_{3,1}\\ {\text{z}}_{3,2,\text{t}}=W_{3,2}{\text{z}}_{2,t}+{\text{b}}_{3,2}\\ \vdots \\ {\text{z}}_{3,N,\text{t}}=W_{3,N}{\text{z}}_{2,t}+{\text{b}}_{3,N}\\ f_{3,1,t}=\text{softmax}\left({\text{z}}_{3,1,\text{t}}\right)\\ f_{3,2,t}=\text{softmax}\left({\text{z}}_{3,2,\text{t}}\right)\\ \vdots \\ f_{3,N,t}=\text{softmax}\left({\text{z}}_{3,N,\text{t}}\right) \end{array} \end{array}$$

Where $W_{3,i}\in R^{3\times h_{2}},{\text{b}}_{3,i}\in R^{3}$ for *i* = 1, …, *N*.

When the class of the *i*^*th*^ ant is *j* we define *e*_*ijk*_ = 1 for *j* = *k* and 0 otherwise. The loss function is modified as below and is simply the sum of the categorical cross entropy for each ant.

$$\begin{array}{c} \begin{array}{c} f_{3,i}\left({\left({\text{z}}_{3,i,t}\right)}_{k}\right)=p_{i,k}\\ \text{Loss}=\sum_{i=1}^{N}\sum_{k=0}^{2}e_{ijk}\text{log}(\frac{1}{p_{i,k}})=\sum_{i=1}^{N}\text{log}(\frac{1}{p_{i,j}}) \end{array} \end{array}$$

Tuning of hyper-parameters were done similarly to that of the individual level model.

Since the softmax function outputs a discrete probability distribution over the *K* classes for each ant we sample from a Multinomial Distribution with corresponding probabilities for each ant in order to obtain simulations. For the individual level model we will have to do this only once whereas for the colony level model we will have *N* such distributions.

We also use MLPs to model *f*_*v*_. For the individual level model we have a bi-variate response *v*_*x*_ and *v*_*y*_- the velocities in the *x* and *y* directions. Our final goal is to build a stochastic movement simulator and in order to achieve this we need to be able to generate stochastic movements. We specify neural networks within a Bayesian setting that will let us sample *v*_*x*_ and *v*_*y*_ from a posterior predictive distribution. See [45] for a detailed explanation of Bayesian Neural Networks (BNN). For the individual level model we define a two layer BNN as follows.

$$\begin{array}{c} \begin{array}{c} {\text{z}}_{1,t}=f_{1}\left(W_{1}{\text{u}}_{t-1}+{\text{b}}_{1}\right)\\ {\text{z}}_{2,t}=f_{2}\left(W_{2}{\text{z}}_{1,t}+{\text{b}}_{2}\right)\\ f_{v}\left({\text{u}}_{t-1}\right)=W_{3}{\text{z}}_{2,t}+{\text{b}}_{3} \end{array} \end{array}$$

Where *W*_1_, *W*_2_ and *W*_3_ are *h*_1_ × *p*,*h*_2_ × *h*_1_ and *h*_3_ × *h*_2_ matrices respectively while **b**_1_, **b**_2_ and **b**_3_ are *h*_1_, *h*_2_ and 2 dimensional vectors respectively. We specify *N*(0, *ν*) as the prior distribution for each element of the weight matrices and bias vectors. Bayesian inference can be done using MCMC [45] or more recent variational inference methods [46, 47]. These methods can be computationally prohibitive. [37] suggested dropout as a Bayesian approximation. They prove that casting dropout in training and testing phases in deep neural networks leads to approximate Bayesian inference. They call their method Monte Carlo dropout (MCdropout). Dropout should be used in the testing phase in addition to the training phase. We use the sum of the mean squared errors of the individual velocities as the loss function. The predictions generated are approximate samples from the posterior predictive distribution [35]. For more details about MCdropout see [37].

We use the keras library in Python to implement MCDropout and apply dropout at each layer in the network. Similarly to the classification models we use a grid search to tune the models.

We extend this model to the colony level by including all velocities in *x* and *y* directions of all ants as the response variable. The response variable is then a 146 dimensional vector.

**Fig 4:**
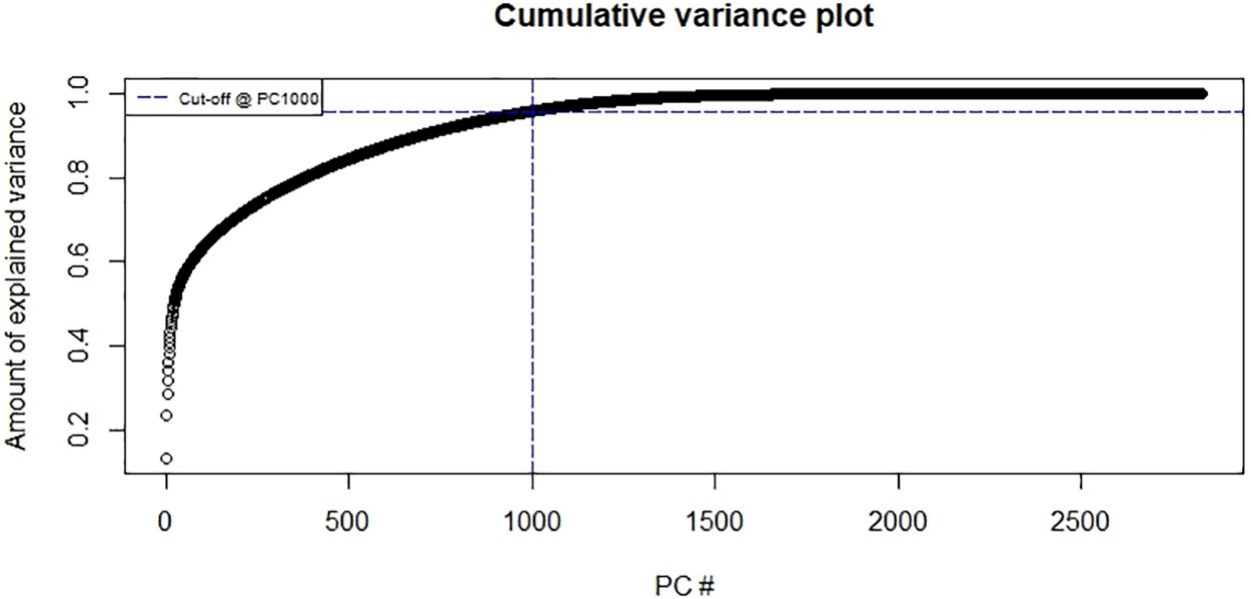
Cumulative variance plot. First 1000 components explain over 95% of the variance.

For feed forward and recurrent neural network models for both movement behaviour and velocity at the colony level we use Principal Component Analysis (PCA) for dimension reduction. PCA has been used as a pre-processing dimension reduction method in [48] and [49]. We extract the first 1000 components which accounts for over 95% of the variability of the input variables. See Fig 4 for the cumulative variance plot.

All other aspects of implementing the colony level NN model for ant velocities were done similarly to the individual level NN model.

#### 2.5.4 Recurrent neural networks

Recurrent Neural Networks(RNN) model sequential information and its output depends on its input as well as past computations. The network thus develops a memory of its past events that are implicitly encoded into its hidden neurons. This differs from traditional feedforward networks where each set of inputs and outputs are independent of each other [50]. RNN and its variants therefore are a popular choice for time series forecasting and some applications include water resources forecasting [51] and electricity spot prices forecasting (Mirikitani and Nikolaev, 2010).

We explore using standard Recurrent Neural Networks for modeling *f*_*m*_ and *f*_*v*_. We consider a two layer recurrent neural network where the first layer is a recurrent layer and the second layer is a feed forward neural network similar to what we defined earlier. The only change for both models *f*_*m*_ and *f*_*v*_ is the form of *f*_1_. This is modified in the following way for a simple RNN [38].

$$\begin{array}{c} {\text{z}}_{1,t}=f_{1}\left(W_{1}{\text{u}}_{t-1}+W_{h}{\text{z}}_{1,t-1}+{\text{b}}_{1}\right) \end{array}$$

Where *W*_*h*_ is a *h*_1_ × *h*_1_ matrix.

An important variant of RNN is long short-term memory (LSTM) [36] which has the ability to retain information for long time intervals. **z**_1,*t*_ is modified as follows

$$\begin{array}{c} \begin{array}{c} i_{t}=\sigma \left(W_{1i}{\text{u}}_{t-1}+W_{hi}{\text{z}}_{1,t-1}+W_{ci}{\text{c}}_{t-1}+{\text{b}}_{1i}\right)\\ f_{t}=\sigma \left(W_{1f}{\text{u}}_{t-1}+W_{hf}{\text{z}}_{1,t-1}+W_{cf}{\text{c}}_{t-1}+{\text{b}}_{1f}\right)\\ c_{t}=f_{t}{\text{c}}_{t-1}+i_{t}\text{tanh}\left(W_{1c}{\text{u}}_{t-1}+W_{hc}{\text{z}}_{1,t-1}+{\text{b}}_{1c}\right)\\ o_{t}=\sigma \left(W_{1o}{\text{u}}_{t-1}+W_{ho}{\text{z}}_{t-1}+W_{co}{\text{c}}_{t-1}+{\text{b}}_{1o}\right)\\ {\text{z}}_{1,t}=o_{t}\text{tanh}\left({\text{c}}_{t}\right) \end{array} \end{array}$$

where *σ* is the sigmoid activation function, *i*, *f*, *o* and *c* are the input gate, forget gate, output gate and cell activation function. All of these are dimension equal to the size of the hidden layer *h*_1_. The input gate *i* is used to protect the memory contents from perturbation of irrelevant inputs. The output gate *o* is similarly used to protect other units from currently irrelevant memory contents. The forget gate was a later addition to the LSTM model [52]. This rectifies a weakness of LSTM that could only handle subsequences with explicitly marked ends and not continual input streams. As specified in section 2.4.2 we used PCA as method of reducing the dimension of the input variables.

In fitting both models we consider the training set as one long time series without dividing the times series into sub-sequences. We use these models for the classification task as well as regression. Both methods have implementations in keras. We use a grid search for model tuning.

### 2.6 SDE model

We have described multiple machine learning methods, which we can use to model animal movement and behavioural states. Most current animal movement research uses parametric models, with one prominent type of model being based on stochastic differential equations. To compare machine learning approaches to parametric models, we will use the ant data and compare results from machine learning methods with a custom stochastic differential equation model developed specifically for our ant system. The stochastic differential equation (SDE) model described in this section assumes constant movement. Thus, we fit the SDE model to the subset of the training data where the ants were in motion. We classified each individual animal movement as “moved within the nest”, “stood still within the nest”, or “outside the nest”, and we used these classes to fit a multinomial regression with backward step selection on the variables in Table 1. When an individual was predicted to have “moved within the nest”, we used the SDE model to predict the next position in time. The SDE model framework [4] captures spatially-varying directional bias using a potential surface and captures spatial variation in speed without directional bias using a motility surface. This SDE model is a custom-constructed parametric model custom-constructed for this ant system. It thus provides a reasonable parametric model for comparison with the nonparametric models we propose.

The SDE model is as described in [53]. The model for animal position **p**_*t*_ at time *t* is represented by the set of equations

$$d{\text{p}}_{t}={\text{v}}_{t}dt$$ (7)

$$d{\text{v}}_{t}=-\beta \left({\text{v}}_{t}-m\left({\text{p}}_{t}\right)\left[-\nabla h\left({\text{p}}_{t}\right)\right]\right)dt+\sigma m\left({\text{p}}_{t}\right)\text{I}dw_{t}$$ (8)

where **v**_*t*_ is the velocity of the animal at time *t*, *β* is the coefficient of friction which controls autocorrelation in movement, *σ* is a constant which we set equal to 1 to render the model identifiable, **I** is a 2 × 2 identity matrix, **w**_*t*_ is independent Brownian motion in $R^{2}$, and *m*(**p**_*t*_) and *h*(**p**_*t*_) are spacially-varying motility and potential surfaces respectively, evaluated at **p**_*t*_. The potential surface captures spatially-varying directional bias (drift) through its gradient, while the motility surface captures spatial variation in speed without directional bias by compressing and dilating time.

The Euler-Maruyama method approximates (7) and (8) by

$${\text{p}}_{t+1}={\text{p}}_{t}+{\text{v}}_{t}$$ (9)

$${\text{v}}_{t+1}={\text{v}}_{t}-\beta ({\text{v}}_{t}-m\left({\text{p}}_{t}\right)\left[-\nabla h\left({\text{p}}_{t}\right)\right]+m\left({\text{p}}_{t}\right)\text{I}dw_{t}$$ (10)

Isolating **v**_*t*_ in (9) and substituting into (10) results in the autoregressive model of order 2

$${\text{p}}_{t+2}=\beta m\left({\text{p}}_{t}\right)\left[-\nabla h\left({\text{p}}_{t}\right)\right]+\left(2-\beta \right){\text{p}}_{t+1}+\left(1-\beta \right){\text{p}}_{t}+m\left({\text{p}}_{t}\right)\epsilon_{t}$$ (11)

where $\epsilon_{t}\overset{\text{iid}}{∼}N\left(0,\text{I}\right)$ and **0** is a column vector of zeroes in $R^{2}$.

Motility and potential surfaces are divided into *J* = 9, 998 1 × 1 mm grid cells. Motility and potential surfaces evaluated at position **p**_*t*_ have the spline representation

$$\begin{array}{c} h({\text{p}}_{t})\equiv \sum_{j=1}^{J}h_{j}s_{j}\left({\text{p}}_{t}\right)\\ m({\text{p}}_{t})\equiv \sum_{j=1}^{J}m_{j}s_{j}\left({\text{p}}_{t}\right) \end{array}$$

where

$$\begin{array}{c} s_{j}\left({\text{p}}_{t}\right)\equiv \{\begin{array}{cc} 1, & {\text{p}}_{t}\;\text{in}\;j^{th}\;\text{grid}\;\text{cell}\\ 0, & \text{otherwise} \end{array} \end{array}$$

and *h*_*j*_ and *m*_*j*_ are the potential and motility surfaces respectively, evaluated in grid cell *j*.

We solved for the motility surface **m**_1:*J*_ by minimizing the penalized likelihood to estimate the variance of the residual, which is proportional to the motility. Plugging in the estimated motility surface, we minimized the penalized likelihood a second time and solved for the potential surface **h**_1:*J*_. This procedure is described in further detail in [53]. Once the motility and potential surfaces were estimated, we made predictions using the autoregressive model (11).

## 3 Results

### 3.1 One step ahead predictions for ant movement

We divide the data into 80% training data and 20% testing data. All models are fitted using the training data and evaluated based on both training and testing data. The mean square prediction error (MSPE) for the training set and testing set is given in Table 2 for each method. Although we model velocities through Eq (2) we compute MSPE based on the predicted (*x*, *y*) locations and the actual location co-ordinates. For locations falling outside of the nest, we project them onto the nearest border of the nest using a vector projection algorithm. See [54] for details. The formula we use for calculating the MSPE is given below. The objective is to have low training and testing MSPE with low disparity between the models. This indicates a high level of accuracy with minimal overfitting of the models to the training set.

$$\begin{array}{c} \text{MSPE}=\frac{\sum_{1}^{n}\sqrt{{\left(x_{t}-\hat{x_{t}}\right)}^{2}+{\left(y_{t}-\hat{y_{t}}\right)}^{2}}}{n} \end{array}$$ (12)

**Table 2:** MSPE for one step ahead predictions for individual and colony level models.

**Individual Movement**		
**Method**	**Train MSPE**	**Test MSPE**
Multinomial Regression + SDE	0.2105	0.2375
Random Forest	0.1765	0.2149
NN	0.1952	0.2375
RNN	0.2057	0.2316
LSTM	0.2060	0.2325
**Colony Movement**		
**Method**	**Train MSPE**	**Test MSPE**
Random Forest	0.2204	0.2522
NN	0.2208	0.2518
RNN	0.2211	0.2519
LSTM	0.2222	0.2543

The individual level Random Forest model had the lowest MSPE for the training and testing sets. The colony level models did worse than the individual level models. When considering only the colony level models the NN model had the best performance for the test set. The RNN model has similar performance to the NN model. The Random Forest at the colony level has low MSPE for the training set but relatively high MSPE for testing set suggesting some degree of overfitting.

We also examined variable importance for the best overall model for one step prediction. The variable importance for models estimating *f*_*m*_ and *f*_*v*_ are given in Table 3.

**Table 3:** Predictor variables with top 5 variable importance for individual level random forest models estimating *f*_*m*_ and *f*_*v*_.

Variable Importance for Movement model (*f*_*m*_)	Variable Importance for Velocity model (*f*_*v*_)
Stationary time	*vy*_*t*−1_
*d*_*t*−1_	*vx*_*t*−1_
*vx*_*t*−1_	Distance to nearest neighbor
*vx*_*t*−2_	*y*_*t*−2_
*vy*_*t*−1_	Distance to wall in East direction

### 3.2 One step ahead predictions for gull migratory movement

Since the purpose of the gull analysis is to demonstrate how this framework can be generally applied to other species we do not apply all the machine learning and deep learning methods given in Section 2.5 but apply only Random Forests and LSTM models. We picked these two since they are the simplest and most complex of the machine learning and deep learning methods we explored.

We randomly split the 15 gull years into 5 folds and used 5-fold cross validation to tune each model using a grid search. For LSTM models, when fitting the velocity models we extracted observations that ended in the specific state we were modeling, and also extracted a moving window of the 1000 prior steps leading up to it. For the Random Forest model this was simpler as it is not recurrent in nature and we simply subset the data by the state we were modeling and used each subset when fitting the velocity models.

In this analysis we let cross validated prediction error for the classification model determine the most appropriate number of lags. We used 3 candidate lags- 30, 40 and 50. For the Random Forest model using 50 lags gave the best performance and therefore we used 50 lags for both the classification and velocity steps. For the LSTM model using a lag of 40 gave the best performance in the classification step and we used 40 lags in the velocity step as well.

We calculated MSPE for each individual fold using the best models from the grid search. We give the mean cross-validated MSPE and corresponding standard deviations in Table 4. The LSTM model does considerably better than the Random Forest model in one step ahead predicitons.

**Table 4:** Average cross-validated MSPE and standard deviations using random forest and LSTM models for migratory gulls.

Method	Average CV MSPE	Standard Deviation of CV MSPE
Random Forest	4.5608	0.1571
LSTM	1.7325	0.1305

We also examined variable importance for the best overall model in predicting one step ahead movement for migratory gulls. Table 5 gives the top five variables for each model (classification and 4 velocity models) in decreasing order. It was surprising that day number did not have a very high variable importance for the classification model although having various lags of *y* is reasonable to expect.

**Table 5:** Variable importance for individual level random forest model in gulls analysis.

Model	Variables with highest importance
Classification	*y*_*t*−9_, *y*_*t*−35_, *y*_*t*−28_, *y*_*t*−15_, *y*_*t*−22_
Velocity for state = 1	*vy*_*t*−1_, *vx*_*t*−1_, *x*_*t*−1_, *y*_*t*−2_, dist
Velocity for state = 2	*vy*_*t*−1_, *vx*_*t*−1_, dist, *vy*_*t*−2_, *vx*_*t*−2_
Velocity for state = 3	*vx*_*t*−1_, *vy*_*t*−1_, *y*_*t*−1_, *y*_*t*−2_,*x*_*t*−2_
Velocity for state = 4	*vy*_*t*−1_, *vx*_*t*−1_, dist, *vy*_*t*−2_, *vy*_*t*−44_

### 3.3 1000 steps ahead predictions for ant movement models

In addition to looking at one step ahead prediction, we also compare the models in Table 2 based on long term simulations. We simulate 1000 steps ahead from the starting point of the testing set. We repeat the simulation 100 times for each model. When comparing these simulations we do not compare prediction accuracy as it is not appropriate when considering stochastic simulations over a long period of time. Rather, we compare features of the simulations of each model. We define metrics to investigate individual behaviour of the ants, space use of the nest and interaction between ants.

When considering individual behaviour we look at “Total Distance” travelled by all the ants over the 1000 step period, we also consider the percentage of time ants are stationary and out of the nest during the time period. When examining space use we look at the percentage of time ants are in each sub chamber (1-8). To investigate interaction between ants we look at the average number of ants within a 12mm radius over the 1000 time step period.

**Fig 5:**
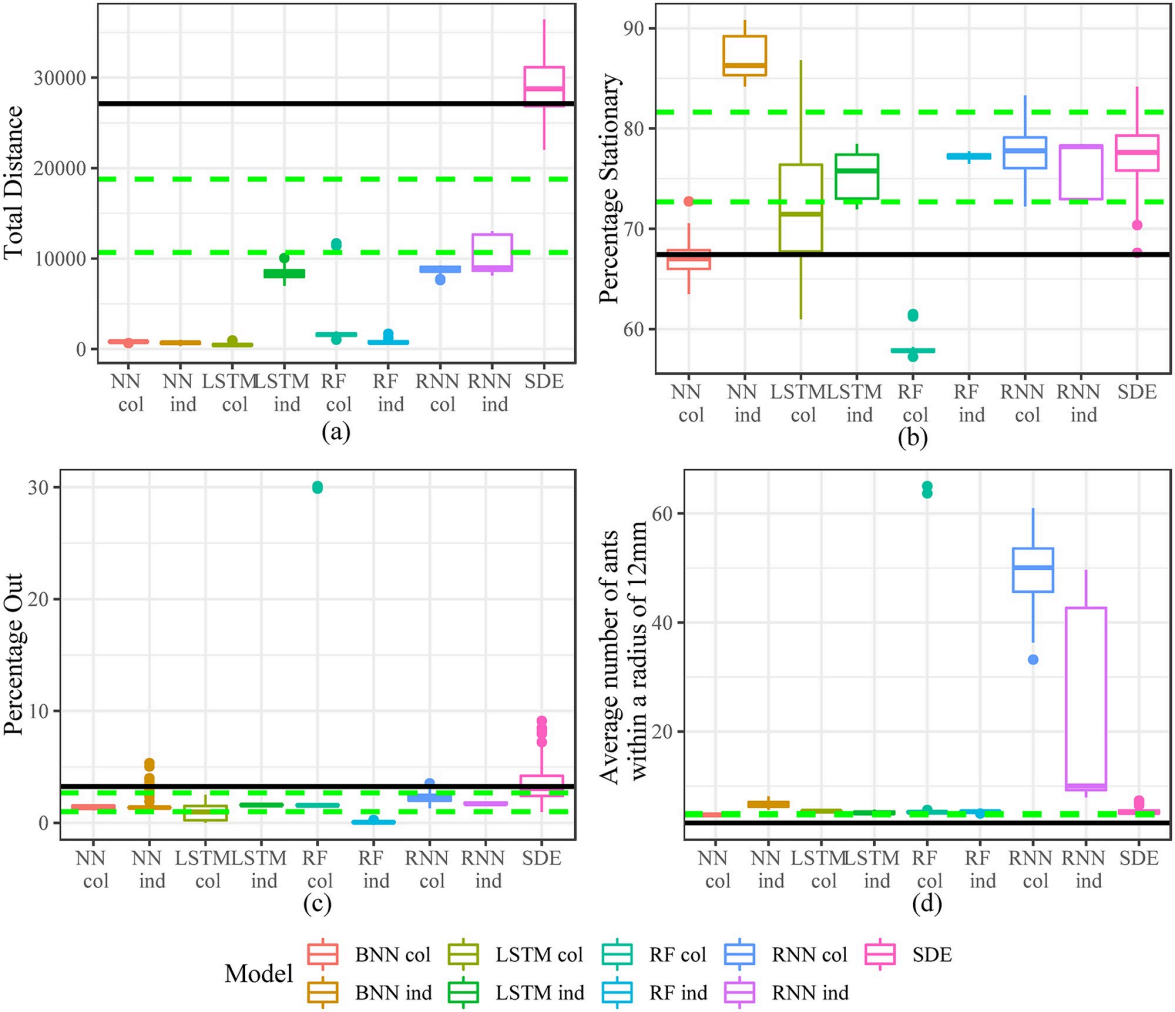
Metrics investigating individual ant behaviour and ant interaction. The black solid line gives the metric for the actual 1000 second time period. The green dashed lines are the 25th and 75th percentiles of the metric for all 1000 second windows in the actual data. (a) Total distance (b) Percentage Stationary (c) Percentage out (d) Average number of ants within a radius of 12mm.

**Fig 6:**
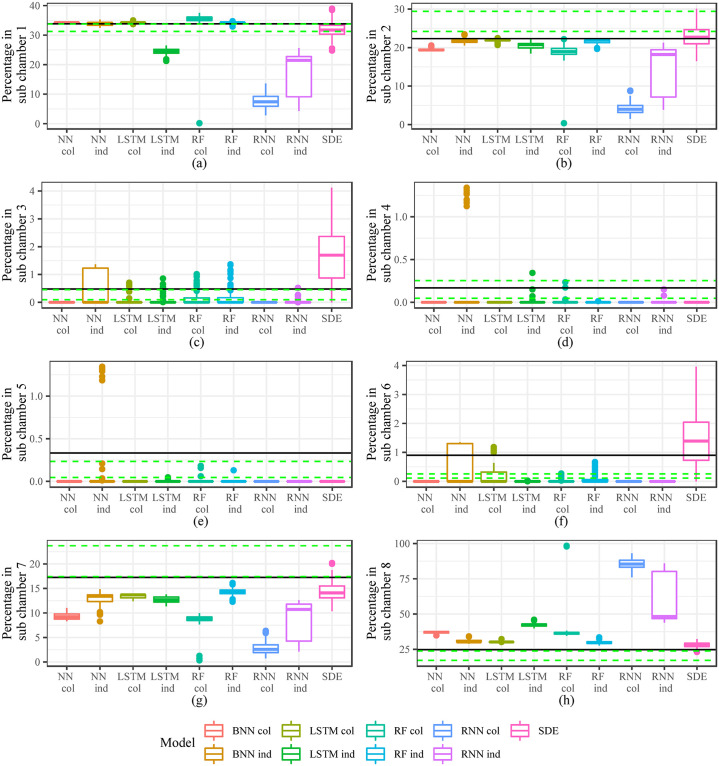
Metrics investigating space use of ants in simulations. The black solid line gives the metric for the actual 1000 second time period. The green dashed lines are the 25th and 75th percentiles of the metric for all 1000 second windows in the actual data. (a) sub chamber 1 (b) sub chamber 2 (c) sub chamber 3 (d) sub chamber 4 (e) sub chamber 5 (f) sub chamber 6 (g) sub chamber 7 (h) sub chamber 8.

We compute these metrics for each of the 100 simulations of each model and compare against the observed metric for the 1000 time step period. Figs 5 and 6 give the results for the 1000 step ahead predictions. The solid black line is the actual metric for the 1000 time step period and the dashed green lines give the 25th and 75th percentiles of a 1000 time step moving window over the whole dataset. This was done to provide a reference as to whether the period we are simulating over is unusual compared to the general movement features we see in the data. We do see that it is a somewhat unusual period as the solid black line falls within the dashed green lines only once (Fig 6 plot (d)) in the 12 plots of Figs 5 and 6.

When looking at Fig 5 we see that the SDE model captures the actual in plots (a) and (c). In plots (b) and (d) the SDE model still is within the dashed green bands. The SDE model struggles somewhat with space use especially plots (c), (d) and (e) of Fig 6.

The machine learning and deep learning models have mixed performance over the different metrics. There is no clear winner. Plot (a) in Fig 5 is of particular concern for these models as many of them seem to have very slight movements. Recurrent models like the LSTM individual level model and the individual and colony level RNN model did better than the other machine learning and deep learning models in plot (a) of Fig 5. The models did better at capturing Percentage out and Percentage stationary, although the NN individual level model and the RF colony level model overestimated and underestimated the metrics respectively for Percentage Stationary. Based on plot (a) of Fig 5 we consider the LSTM individual model, RNN individual and colony models to be contenders of the best machine learning and deep learning models. The RNN colony level model overestimates the metric significantly in plot (d) of Fig 5 while the LSTM individual model has better performance than the RNN individual model in all the plots of Fig 6.

None of the models were able to simulate a movement path from sub chamber 1 through to sub chamber 8 or in the opposite direction.

Overall, the SDE model did best in capturing the features of ant movement. From the machine learning and deep learning models the LSTM individual level model seems to have the most reasonable performance in long range simulations.

### 3.4 Simulated migratory paths using gull models

We do not conduct a comprehensive simulation study for the gull models like we did in section 3.3 for the ant models. This is because our purpose in doing the analysis for the gulls data is to demonstrate the generalizability of the framework we propose. Instead we present simulated migratory paths for Random Forests and LSTM models for illustrative purposes. We randomly selected a gull year (gull ID 325 from year 2010) for the simulations. We refitted the models in Table 4 excluding the randomly selected gull year.

**Fig 7:**
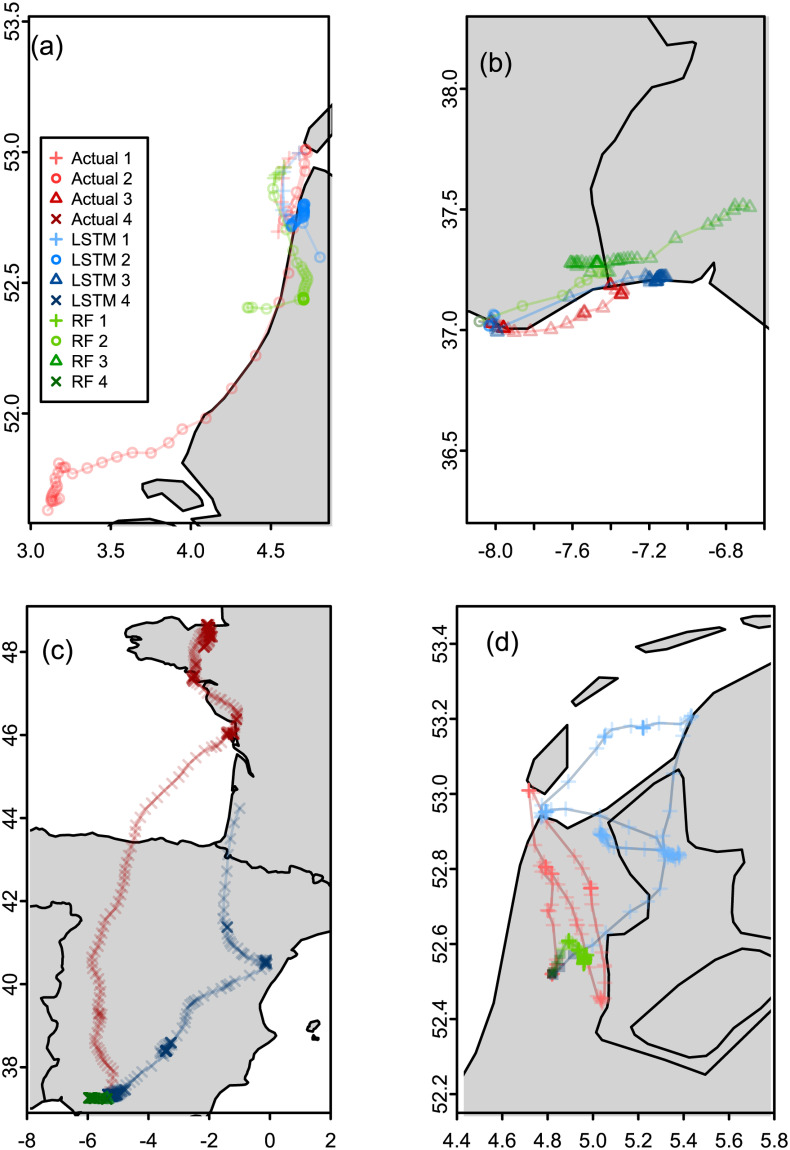
Simulation of shorter migratory paths transitioning from one state to another. (a) Transition from state 1 to state 2 (b) Transition from state 2 to 3 (c) Transition from state 3 to 4 (d) Transition from state 4 to 1. Red is the actual migratory path, green is the path simulated using the LSTM and blue is the path simulated using the Random Forest model. The color gradient for each color denotes the different states as given in the plot legend.

**Fig 8:**
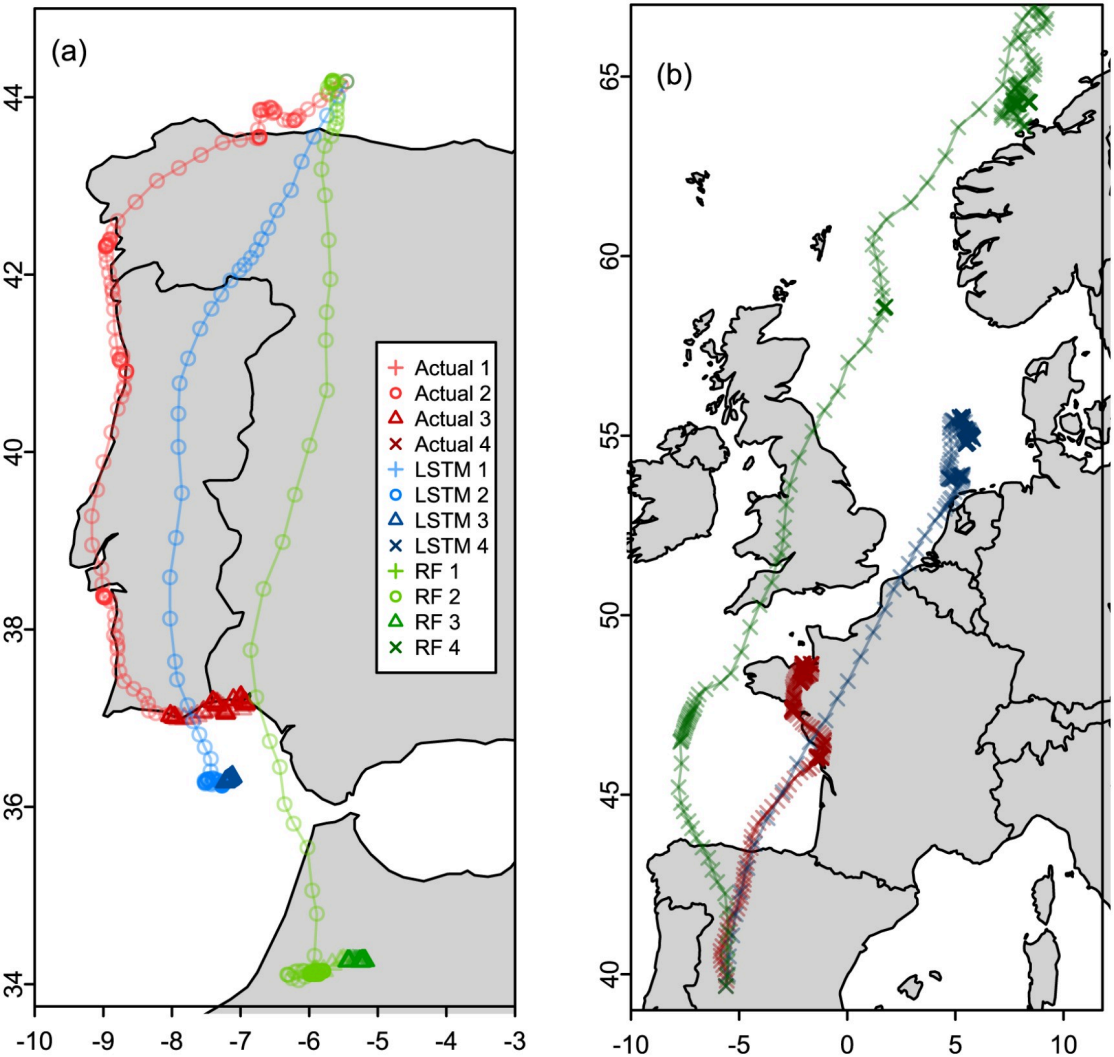
Simulation of shorter migratory paths within the Southern and Northern migration states. (a) Southern migration 2 (b) Northern migration. Red is the actual migratory path, green is the path simulated using the LSTM and blue is the path simulated using the Random Forest model. The color gradient for each color denotes the different states as given in the plot legend.

Simulating complete annual paths of gull migration were not successful therefore we simulated shorter (50 to 300 steps ahead) simulation paths, focusing on the transition points of one state to another (Fig 7) as well as within the Southern migration and Northern migration states (Fig 8).

Paths given in red are the actual paths, the paths in blue and green are the simulated paths from the LSTM and Random Forest models respectively. All the simulated paths in Fig 7 captured the change in state as evidenced in the subtle change in the darkness/lightness of the color of the path. Fig 7(a) shows both the LSTM and RF simulations transitioning to state 2 and moving southward although they do not move as much as the actual path given in red. In Fig 7(b) both the LSTM and RF simulated paths transition to state 3, the LSTM simulation gives the gull hovering on the coast like the actual path while the RF simulation gives the gull moving more inland. In Fig 7(c), although both the LSTM and RF transition to state 4 which is Northern migration, the Random Forest model does not move in the Northern direction instead moving slightly towards the West. The LSTM model however shows migration to the North. Fig 7(d) shows both the LSTM and RF paths transitioning to state 1. The RF path has very small steps while the LSTM path has movement that visually looks more similar to the actual path.

Fig 8 gives simulated paths of gulls while the gulls are in their migratory states. Fig 8(a) shows both the LSTM and RF paths staying in state 2 while moving southwards and eventually transitioning to state 3 similar to the actual path. Both the simulated paths show the transition to state 3 happening further south than the actual path with the RF path ending in Africa. Fig 7(b) show both the LSTM and RF model continuing in state 4 and moving in the Northern direction. The RF simulated path moves a lot more further North than the actual path.

Overall, looking at the simulated paths given in Figs 7 and 8, the LSTM simulated paths capture the qualitative nature of the actual movement. Code used in this study can be found at https://github.com/dzw5320/animalmov.

## 4 Discussion

In this study we proposed a general framework to predict and simulate animal movement. We applied this framework using various machine learning and deep learning models as well as a custom constructed parametric movement model for the ant data. We also demonstrated the generalizeability of our approch by applying this framework to the gull data at the individual level. For the ant data, we found that for one step ahead prediction the machine learning and deep learning models at the individual level did better than the SDE model. The random forest model at the individual level had the best performance for one step ahead prediction. For the gull data, the LSTM model did better than the Random Forest model when predicting one step ahead. For ant movement simulation, the SDE model seemed to do better than the other models although none of them were a clear winner. Of the machine learning and deep learning models the LSTM individual level model had the overall best performance when considering simulations. For the simulated gull migratory paths, the LSTM model seemed to better capture migratory behaviour than the random forest model.

The general framework has two main steps. In the first step we predict the movement behaviour of the animal. Given the movement behaviour, we then predict the bivariate velocity. When fitting the second step for the Random Forest and Neural Network models at the individual level we only used the data where an animal made a movement to fit the velocity model. For the RNN and LSTM models this was more complex, and for the ant data, for ease we used all the data irrespective of whether the movement resulted in the ant moving or not to fit the velocity model. An alternative method would be to only consider sequences of movements that end with the animal in the state under consideration. We followed this approach when fitting the LSTM model for the gull data. This leads to the data in the training set having variable time lengths. However, this can be handled by padding the time sequences and indicating that padding has been used when specifying the model in keras.

In the ant analysis, the colony level models had deteriorated performance when compared to the individual level models. Here again we used all the data to fit the velocity model. Unlike for RNN and LSTM models in the individual level there is no alternative method to only subset for when the ants are moving as at any given time some ants are stationary while others move. Here we fit two independent models for movement behaviour and velocity and then combine their outputs by zeroing out velocities if the ants are predicted to be stationary or making the appropriate velocity adjustment if the ant is predicted to move out of the nest.

A possible reason for the performance of using colony level models suffering could be the low volume of data. Since we are modeling all the ants together the number of samples we have now reduces to the number of time points in the training data set for non recurrent models and to one long sample for the recurrent neural networks. The number of predictor variables increase thereby increasing the size of the network. These two factors in conjunction lead to difficulties in the colony level model. Machine Learning and deep learning methods typically need large amounts of data. We used truncated Principal Component Analysis as a dimension reduction method for the predictor variables. This method was chosen due to ease of implementation and its simplicity. Alternative dimension reduction can be used here. A drawback of using PCA is that the already difficult problem of investigating variable importance now gets more difficult since each component is a mixture of the original predictor variables.

Most colonies have social hierarchies which delineate roles to each animal that in turn could affect their movement behaviour. Even within the ant colony, we typically see a queen, workers and males. It might be useful to first use clustering to separate each type of animal and then apply the framework proposed in this study so that there is greater homogeneity in the data used to fit each model.

We computed variable importance for the best model for one step ahead prediction in the ant analysis which was the Random Forest model at the individual level. We did this for the gull random forest model as well. Computing variable importance for Random Forests models is a well studied problem (see [55] and [56]). Computing variable importance for neural networks is less straightforward. [57] suggested conducting a functional analysis of the weight matrix based on a technique that determines behavioral significance of hidden neurons. Another method is to consider the neural network to be a black box and use permutation importance as given in [56] to calculate variable importance. In our case we could consider the truncated PCA as part of the black box.

The ease of specifying machine learning and deep learning models compared to traditional animal movement models is an important factor to consider. Most traditional parametric models are custom constructed for specific species or environments. Therefore developing a model can be time consuming. There are also various assumptions made in specifying these models which might not be accurate or realistic across various scenarios. Machine Learning and Deep Learning models are easier to specify and fit although tuning these models can often be laborious. There has been a significant improvement in the computing capability of the processing units that are used to run these models. They are also readily available for use via cloud computing [58]. This increase in computing capability has also meant that they scale well to large amounts of data. However, the model used need to depend on the goal of the study. If prediction or simulation is the goal, machine learning or deep learning methods might be easier to specify due to the black box nature of these methods and faster to fit due to advanced computing resources that are now available [58]. This comes at the cost of the models being less interpretable.

Tuning a neural network can often be tedious. There are a number of tuning parameters that need to be specified. For e.g. the number of hidden layers, the number of neurons in each hidden layer, the optimizer to use, the activation function, the level of dropout, the learning rate and the number of iterations used to train the model. In addition to these there are additional tuning parameters that help in avoiding over-fitting like L1 and L2 regularization. We used a grid search to train the neural network. Another method that is gaining popularity for tuning is using genetic algorithms [59, 60].

This study introduces a novel framework for classification of movement states and predicting movement velocities. This is particularly useful when it is straightforward to identify movement behaviour states and data is available at a fine temporal resolution. This might not always be the case. If data is sparse in time and states are not easily identifiable you will need to identify states before the analysis as we did in the gull analysis. Some degree of pre-processing could be used for this. There are existing frameworks like [61] that are useful for going from sparse data to fine-resolution markov chains.

Reinforcement learning is a field of artificial intelligence that has been used for problems like modeling self driving cars [62, 63]. It will be interesting to see if formulating the simulation problem through this paradigm would give better performance.

We present a general framework for modeling animal movement that consists of two steps. The first step models movement behaviour and the second step models velocity of the animal given the movement behaviour. We used a suite of machine learning and deep learning to fit individual and colony level models. We use ant movement data and compare performance against stochastic differential equation model that uses multinomial regression to model movement behaviour. Random Forests and Neural Networks at the individual level perform better than the SDE model for one-step ahead predictions while the SDE model does better in capturing movement features in long range simulations. The LSTM individual level model has the best performance out of the machine learning and deep learning models for long range simulations of ant colonies. In order to demonstrate the utility of the framework generally, we also applied the Random Forest and LSTM models to gull movement data at the individual level and found that the LSTM model had better performance for one step ahead predictions as well as better visual simulated paths. Machine Learning and Deep Learning have been sparingly used to model movement behaviour or predicting locations. This work provides a unified approach of combining both aspects within a general framework.
